# Limited Long‐Range Olfactory Discrimination in a MATOPIBA Lineage of *Encarsia* (Hymenoptera: Aphelinidae) Across Resistant and Susceptible Crop Cultivars

**DOI:** 10.1002/arch.70234

**Published:** 2026-11-15

**Authors:** Daniel Marques Pacheco, Maria Carolina Farias e Silva, Adriele Castro Ferreira, Matheus Monteiro de Santana, Rodolfo Molinário de Souza, Jenilton Gomes da Cunha, Alexandre Igor de Azevedo Pereira, Bruno Ettore Pavan, José Bruno Malaquias, Ana Paula Gonçalves da Silva Wengrat, Luciana Barboza Silva

**Affiliations:** ^1^ Federal University of Piauí Professora Cinobelina Elvas Campus Bom Jesus Brazil; ^2^ Federal Institute of Education Science and Technology of Goiás, Urutaí Campus Urutaí Brazil; ^3^ São Paulo State University “Júlio de Mesquita Filho” (UNESP) Ilha Solteira Campus Ilha Solteira Brazil; ^4^ Federal University of Paraíba Center for Agricultural Sciences Areia Brazil; ^5^ Department of Entomology and Acarology, “Luiz de Queiroz” College of Agriculture University of São Paulo (ESALQ) Piracicaba Brazil

**Keywords:** *Encarsia*, herbivore‐induced plant volatiles, olfactory responses, parasitoid foraging behaviour, Plant resistance, Tritrophic interactions

## Abstract

Herbivore‐induced plant volatiles (HIPVs) are key mediators of tritrophic interactions, frequently guiding parasitoid host‐searching behaviour over long distances. However, the reliability and discriminatory power of these cues may depend on plant resistance traits and herbivore identity. Here, we evaluated whether volatile emissions from resistant and susceptible soybean and common bean cultivars infested by the whitefly *Bemisia tabaci* MEAM1 or the aphid *Aphis craccivora* influence the long‐range foraging responses of an undescribed *Encarsia* lineage from the MATOPIBA region (Brazil). Using Y‐tube olfactometer bioassays, we recorded initial choice and residence time of individual females exposed to contrasting plant–herbivore combinations. Choice frequencies were analyzed using chi‐square tests, and residence time using non‐parametric analyses, including paired comparisons between olfactometer arms within each contrast. Across all contrasts, parasitoids showed no significant discrimination between resistant and susceptible cultivars, between host plant species, or between plants infested by different herbivores. Residence time did not differ among treatments or between arms within contrasts. These results suggest that, under the experimental conditions tested (plant‐*vs.*‐plant contrasts without a positive control), volatile blends emitted by these crop systems did not provide sufficiently contrasting long‐range cues to influence parasitoid orientation. The observed behavioural pattern is consistent with a generalist long‐range foraging strategy in which host location may depend more strongly on short‐range or contact‐mediated signals. Our findings highlight context‐dependent limits of volatile‐mediated signalling in agroecosystems and contribute to understanding how plant resistance and herbivore identity interact with parasitoid sensory ecology.

## Research Highlights

- The MATOPIBA *Encarsia* lineage showed no long‐range olfactory discrimination among resistant and susceptible crop cultivars.
- Host plant species and herbivore identity did not significantly influence parasitoid orientation behaviour.
- Results suggest that this Encarsia lineage may rely primarily on short‐range or contact‐mediated host‐location cues.

## Graphical abstract

Herbivore‐induced plant volatiles (HIPVs) emitted by resistant and susceptible soybean and common bean cultivars infested with *Bemisia tabaci* MEAM1 or *Aphis craccivora* were evaluated for their effects on the foraging behaviour of *Encarsia* sp. in a Y‐tube olfactometer. Parasitoids showed no preference among plant cultivars, host plant species, or herbivore‐induced volatile blends, indicating that these HIPVs did not provide reliable long‐range cues for host location.

## 1 Introduction

Herbivore‐induced plant responses play a fundamental role in structuring multitrophic interactions by mediating information flow between plants, herbivores, and their natural enemies (Takabayashi 2022). Among these responses, herbivore‐induced plant volatiles (HIPVs) frequently function as long‐range chemical signals that guide parasitoid host‐searching behaviour. However, the ecological reliability of HIPVs as orientation cues depends on their intensity, chemical distinctiveness, and contextual stability. Variation in plant resistance traits, herbivore identity, and environmental conditions may alter volatile blends, potentially reducing their discriminatory power for foraging parasitoids (Silva et al. 2025). Understanding when volatile cues fail to elicit behavioural discrimination is therefore central to parasitoid sensory ecology and to the adaptive dynamics of tritrophic systems.

The whitefly *Bemisia tabaci* MEAM1 (Hemiptera: Aleyrodidae) provides a robust model for investigating plant–herbivore–parasitoid interactions in agroecosystems. This globally distributed and highly polyphagous species causes substantial economic losses through direct phloem feeding and indirect effects such as virus transmission and honeydew deposition (De Barro et al. 2011; Cruz and Baldin 2017; Li et al. 2021; Schutze et al. 2022). The genus *Encarsia* (Hymenoptera: Aphelinidae) comprises key natural enemies of whiteflies and plays a major role in biological control programmes. Although some species, such as *E. formosa*, have demonstrated high parasitism rates under favourable conditions (Yang et al. 2022), parasitoid efficiency is highly context‐dependent and influenced by plant traits, herbivore assemblages, and sensory constraints. Volatiles released by whitefly‐infested plants are known to elicit host‐locating responses in *E. formosa* (Birkett et al. 2003; Ayelo et al. 2021), but whether other, less studied *Encarsia* lineages respond similarly remains largely unknown.

The *Encarsia* population investigated here belongs to an undescribed lineage provisionally referred to as *Encarsia* sp. MATOPIBA lineage, after the agricultural frontier of Brazil formed by the states of Maranhão, Tocantins, Piauí and Bahia. Morphological and molecular evidence indicates that this population does not correspond to any formally described species and is currently under taxonomic characterization. Behavioural divergence among closely related *Encarsia* species is well documented, suggesting that lineage‐specific sensory and foraging strategies may shape ecological outcomes. Studying this lineage therefore offers an opportunity to evaluate how parasitoid behavioural responses operate in a rapidly expanding tropical agroecosystem.

Plant resistance traits can modify herbivore performance and feeding patterns, thereby influencing the quantity and composition of HIPVs. Such changes may enhance, weaken, or qualitatively alter parasitoid attraction (de Rijk et al. 2013). Moreover, herbivore identity and density can further modulate volatile emission patterns, sometimes generating chemically overlapping or weakly induced blends that reduce long‐range detectability (Ayelo et al. 2021). When volatile cues lack sufficient contrast, parasitoids may adopt flexible or generalist strategies, relying more heavily on short‐range sensory inputs such as visual stimuli, honeydew cues, or contact chemicals during host localization (Chen et al. 2020; Ayelo et al. 2022).

In this study, we examined whether plant resistance status and herbivore identity influence the long‐range foraging behaviour of a MATOPIBA *Encarsia* lineage. Using resistant and susceptible cultivars of soybean and common bean infested by *B. tabaci* MEAM1 or the non‐host aphid *Aphis craccivora*, we tested three hypotheses: (i) variation in plant resistance alters parasitoid olfactory discrimination; (ii) host plant species modulates parasitoid orientation; and (iii) herbivory by a non‐host insect modifies parasitoid responses to host‐infested plants. By evaluating these contrasts under controlled olfactometer conditions, we aim to clarify context‐dependent limits of volatile‐mediated host searching and to advance understanding of parasitoid behavioural adaptation in crop‐based tritrophic systems.

## 2 Materials and Methods

### 2.1 Study Site and Environmental Conditions

Bioassays were conducted at the Plant Propagation Laboratory, Federal University of Piauí, Professora Cinobelina Elvas Campus (9°05′02.9″ S; 44°19′34.2″ W; 270 m a.s.l.), Bom Jesus, Piauí, Brazil. Experiments were performed from January to June (regional rainy season) under controlled laboratory conditions (28 ± 2°C; 60–70% relative humidity). Insect colonies and plants were maintained under natural photoperiod conditions (~13 h light:11 h dark), consistent with seasonal daylight patterns of the Southern Hemisphere.

### 2.2 Plant Material

Soybean (*Glycine max* L.) and common bean (*Phaseolus vulgaris* L.) cultivars exhibiting contrasting resistance levels to *Bemisia tabaci* MEAM1 were used (Table 1). Resistance classification (antixenosis and/or antibiosis) was based on previously published studies. Plants were grown individually in 5‐L pots containing a 1:1:1 mixture of soil, washed sand, and cattle manure. Plants used in bioassays were standardized at phenological stages V3–V4 to minimize ontogenetic variation in volatile emission.

**Table 1:** Soybean and common bean cultivars used in the study, their respective breeding companies, and documented resistance to *Bemisia tabaci* MEAM1.

Crop	Cultivar	Company	Resistance type	References
Soybean	BRASMAX BÔNUS IPRO	Brasmax	Susceptible	Rodrigues et al. (2021); Silva et al. (2023)
Soybean	M 8808 IPRO	Monsoy	Antixenosis and Antibiosis (Resistant)	Rodrigues et al. (2021); Silva et al. (2023)
Common Bean	PÉROLA	Embrapa	Susceptible	Peixoto and Boiça Júnior (2014)
Common Bean	BRS ESTILO	Embrapa	Antixenosis and Antibiosis (Resistant)	Santos et al. (2021)

### 2.3 Insect Colonies

#### 2.3.1 *Bemisia tabaci* MEAM1

Whiteflies were initially collected from insecticide‐free tomato crops and maintained on kale (*Brassica oleracea* var. *sabellica*) under greenhouse conditions. Plants were cultivated in 5‐L pots with the same substrate mixture described above. The greenhouse was covered with transparent plastic film and shade netting, and enclosed with 50‐mesh anti‐aphid screen. Colonies were maintained under continuous generation turnover. Biotype identity (MEAM1) was confirmed by molecular analysis performed at Embrapa Rice and Beans.

#### 2.3.2 Aphis craccivora

The aphid colony was established from individuals collected in common bean fields in Bom Jesus, Piauí, Brazil, and maintained on potted common bean plants under greenhouse conditions as described above. Colonies were refreshed every 10–15 days by transferring individuals to new plants to prevent overcrowding and maintain colony vigour.

#### 2.3.3 *Encarsia* sp. (MATOPIBA lineage)

The parasitoid colony originated from *B. tabaci* nymphs collected from kale plants maintained at the Federal University of Piauí. Parasitoids were reared following an adaptation of Generoso (2006), with kale plants bearing whitefly nymphs exposed to parasitism. Rearing on kale, rather than on the crop species used in the bioassays, was adopted to avoid pre‐exposure of the parasitoids to the test‐plant volatiles; consequently, females had no previous experience with soybean or common bean odours before testing. Newly emerged adults were collected daily from the rearing cages and kept in ventilated cages with honey as food; females were used in bioassays when 24–48 h old and were assumed to be mated because they were held together with males. Females were naïve (without previous oviposition experience) at the time of testing.

Specimens were examined morphologically and molecularly at the Biological Institute of São Paulo. Morphological analyses indicated that this population does not correspond to any formally described species of *Encarsia* currently known from the Neotropical region.

For molecular characterization, genomic DNA was extracted following the protocol proposed by Gilbert et al. (2007), adapted for micro‐hymenopterans according to Wengrat et al. (2021). Fragments of the mitochondrial cytochrome c oxidase subunit I gene (COI) and the D2 expansion region of the 28S rDNA gene (28S‐D2) were amplified.

The COI fragment was amplified using the forward primer C1‐J‐2183 and reverse primer TL2‐N‐3014 (Simon et al. 1994), whereas the 28S‐D2 region was amplified using primers D2F and D2R (Campbell et al. 1993). PCR conditions followed Gariepy et al. (2014), with annealing temperatures adjusted to 50°C for the 2183/3014 primer pair and 55°C for D2F/D2R.

Amplicons were visualized under ultraviolet light following electrophoresis in 1.5% agarose gel stained with SYBR Safe. PCR products were purified using Exonuclease I (Exo I) and Shrimp Alkaline Phosphatase (rSAP) enzymes and subsequently sequenced using the Sanger method by ACTgene (Brazil).

Chromatograms were analyzed in Geneious Prime 2022.1 (Biomatters Ltd., Auckland, New Zealand) to verify sequence quality and edit low‐quality regions. Four COI sequences (752 bp) and two 28S‐D2 sequences (651 bp) were obtained and compared, using BLASTn, with all sequences of *Encarsia* species available in GenBank (NCBI) to assess phylogenetic affinity and molecular divergence relative to described *Encarsia* species; none of the described species represented in GenBank matched the sequences obtained. The sequences generated in this study were deposited in GenBank under accession numbers PZ549164–PZ549167 (COI) and PZ554278–PZ554279 (28S‐D2).

Combined morphological and molecular evidence indicates that the studied population represents an undescribed lineage currently under formal taxonomic characterization. Voucher specimens from the field collection and from the laboratory rearing colony (three females and one male, slide‐mounted) were deposited in the Oscar Monte Entomophagous Insect Collection, Biological Institute, Campinas, São Paulo, Brazil, under accession numbers IB‐CBE 008516, IB‐CBE 008991 and IB‐CBE 008992 (females) and IB‐CBE 008993 (male).

### 2.4 Herbivore Infestation and Standardization

For *B. tabaci* treatments, approximately 100 unsexed adults were confined on each plant using voile cages for 12 days prior to bioassays, allowing oviposition and establishment of immature stages. Adults were then removed from the plants, so that only eggs and nymphs (predominantly third–fourth instars) were present at the time of the bioassays. For *A. craccivora* treatments, 20 apterous adults were confined on each plant in ventilated glass cages for 6 days to ensure active feeding and induction of plant responses.

To reduce variability among replicates, plants were selected based on visual confirmation of active herbivore establishment and comparable levels of infestation prior to bioassays. Only plants presenting uniform signs of feeding activity (presence of nymphs and/or aphid colonies distributed across fully expanded leaves) were used in assays. Plants showing atypical infestation patterns or signs of stress unrelated to herbivory were discarded.

All plants were standardized at phenological stages V3–V4 and similar vegetative size to minimize ontogenetic and morphological variation in volatile emission.

Each contrast consisted of 60 independent replicates (Table 2).

**Table 2:** Combinations of host plants infested by *Bemisia tabaci* MEAM1 and *Aphis craccivora* used in the experimental contrasts conducted in a Y‐tube olfactometer to evaluate the behaviour of *Encarsia* sp.

Combinations	Arm A	Arm B
T1	Resistant soybean (M8808) infested with *B. tabaci*	Susceptible soybean (Bônus) infested with *B. tabaci*
T2	Susceptible common bean (Pérola) infested with *B. tabaci*	Resistant common bean (Estilo) infested with *B. tabaci*
T3	Susceptible soybean (Bônus) infested with *B. tabaci*	Susceptible common bean (Pérola) infested with *B. tabaci*
T4	Susceptible common bean (Pérola) infested with *A. craccivora*	Susceptible common bean (Pérola) infested with *B. tabaci*

### 2.5 Y‐Tube Olfactometer Setup and Bias Control

Behavioural assays were conducted in a glass Y‐tube olfactometer (Figure 1) consisting of a main arm (5.5 cm long) and two lateral arms (5.5 cm long, forming an angle of 66°), all with an internal diameter of 4.0 mm. Purified air was supplied by a PVAS22 air‐delivery pump (Volatile Assay Systems, Rensselaer, NY, USA) fitted with hydrocarbon (activated‐charcoal) filters, humidified by bubbling through distilled water and conducted through PTFE tubing at 2.0 L min^−1^ into each odour‐source chamber. The air leaving each chamber was conducted through polyethylene tubing to the corresponding olfactometer arm at 0.4 L min^−1^, regulated by flowmeters, and a suction line connected to the base of the main arm removed air from the system at the same rate to ensure continuous circulation (Figure 1). Flow symmetry between arms was verified with the flowmeters prior to each experimental session. The olfactometer was positioned horizontally on a white bench under artificial illumination (2000 lux), and assays were run between 08:00 and 16:00 h at 28 ± 2°C and 60%–70% relative humidity.

**Figure 1:**
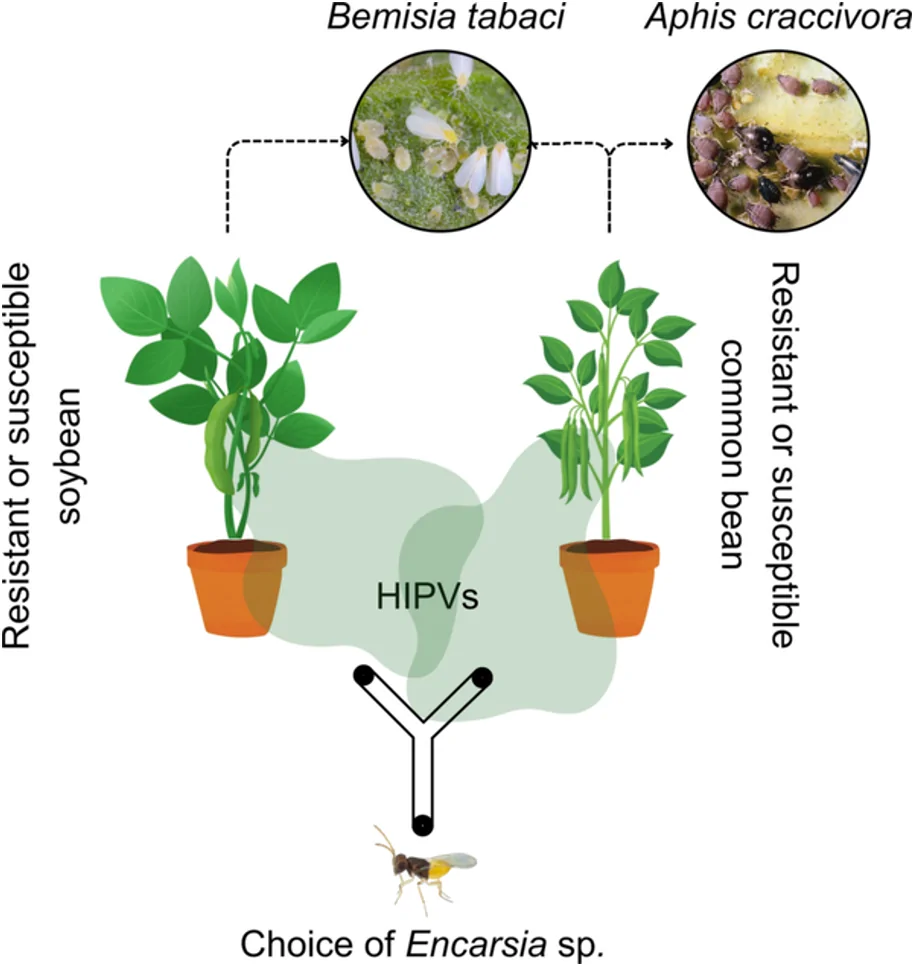
Schematic representation of the Y‐tube olfactometer assay. Each treatment consisted of 60 replicates. T1: resistant versus susceptible soybean infested with *Bemisia tabaci*; T2: susceptible versus resistant common bean infested with *B. tabaci*; T3: susceptible soybean versus susceptible common bean, both infested with *B. tabaci*; T4: common bean infested with *Aphis craccivora* versus *B. tabaci*.

Odour sources consisted of individual infested plants placed in sealed acrylic chambers (0.50 × 0.30 m, with a central opening of 19 cm in diameter) connected to the olfactometer arms as described above. Before the aerial part of each plant was introduced into the chamber, the pot and soil were sealed with aluminium foil to prevent soil‐derived odours from contaminating the airflow. Each pair of plants was used for six replicates and then replaced by a new pair to avoid potential odour saturation or depletion effects.

To minimize directional bias, the odour sources were switched between the olfactometer arms after every three replicates, airflow balance between the arms was verified before each replicate, and the Y‐tube was cleaned with neutral detergent, rinsed with distilled water, and oven‐dried between experimental blocks. Parasitoids were tested individually, and each female was used only once.

Before the treatment bioassays, a preliminary blank test (clean air vs. clean air; *n* = 60 females) was performed to confirm that the olfactometer had no intrinsic arm or positional bias. Twenty‐two females (36.7%) made a choice, whereas 38 (63.3%) did not cross the decision line within 15 min. Responders were evenly distributed between arms (arm A: 8; arm B: 14; *χ* ^2^ = 1.64, df = 1, *p* = 0.20), and residence time did not differ between arms (Wilcoxon signed‐rank test, *p* = 0.70, *r* = 0.08), confirming that the apparatus did not bias parasitoid orientation. Because the study aimed to compare the relative attractiveness of paired plant–herbivore combinations, all treatment contrasts were designed as plant‐versus‐plant comparisons and a positive control (infested plant vs. clean air) was not included; the implications of this design are considered in the Discussion.

### 2.6 Behavioural Recording Criteria

Each female was individually introduced at the base of the main arm of the olfactometer using a fine brush and observed continuously for 15 min. A choice was recorded when the parasitoid crossed a predefined decision line located 2 cm from the Y‐junction and remained in that arm for at least 10 s. Residence time was calculated as cumulative time spent in each arm and in the central zone during the 15‐min observation period.

Individuals who did not cross the decision line within the observation period were classified as “no choice”. The proportion of no‐choice individuals was recorded to ensure that inactivity levels did not differ systematically among treatments.

### 2.7 Statistical Analysis

All analyses were performed in R (v4.5; R Core Team 2025). Figures were generated using SigmaPlot (v14.5). Statistical significance was set at α = 0.05.

Choice data: For each treatment contrast, parasitoid choice frequencies were analyzed using Pearson's chi‐square test to assess deviation from an expected 1:1 distribution under the null hypothesis of no directional preference.

Effect sizes were estimated using Cramér's V to quantify the magnitude of treatment effects independently of sample size. This metric allowed evaluation of whether non‐significant results reflected biologically negligible effects rather than insufficient statistical power.

Individuals classified as “no choice” (i.e., those that did not cross the predefined decision line within 15 min) were excluded from directional preference analyses because they did not express active orientation behaviour. However, the proportion of no‐choice individuals was recorded for each treatment and compared across contrasts using a chi‐square test of homogeneity to assess whether responsiveness differed among experimental conditions.

Residence time data: Residence time (cumulative time spent in each arm and in the central zone) was analyzed using the Kruskal–Wallis test following detection of non‐normal distributions (Shapiro–Wilk test, *p* < 0.05). When appropriate, pairwise comparisons were conducted using Dunn's test with Bonferroni correction.

Effect sizes for non‐parametric analyses were calculated using eta‐squared (*η* ^2^), providing an estimate of the proportion of variance explained by treatment effects. This approach enabled assessment of biological relevance even in the absence of statistical significance.

Central‐zone residence time was analyzed separately to evaluate exploratory or indecisive behaviour independent of directional preference.

In addition, to evaluate whether females allocated more time to one of the two odour sources after entering the arms, residence time in arm A was compared with residence time in arm B within each treatment combination using the Wilcoxon signed‐rank test for paired samples, since both values were obtained from the same individual. Effect sizes for these paired comparisons were expressed as *r* = *Z*/√*N*.

Independence and replication: Each parasitoid female was tested only once, and each replicate was conducted using independent plant material. Treatment positions were alternated systematically to prevent positional bias. These procedures ensured statistical independence and minimized pseudo‐replication.

## 3 Results

### 3.1 Choice Responses

In Y‐tube olfactometer assays, no significant directional preference was detected in any of the evaluated contrasts (Figure 2).

**Figure 2:**
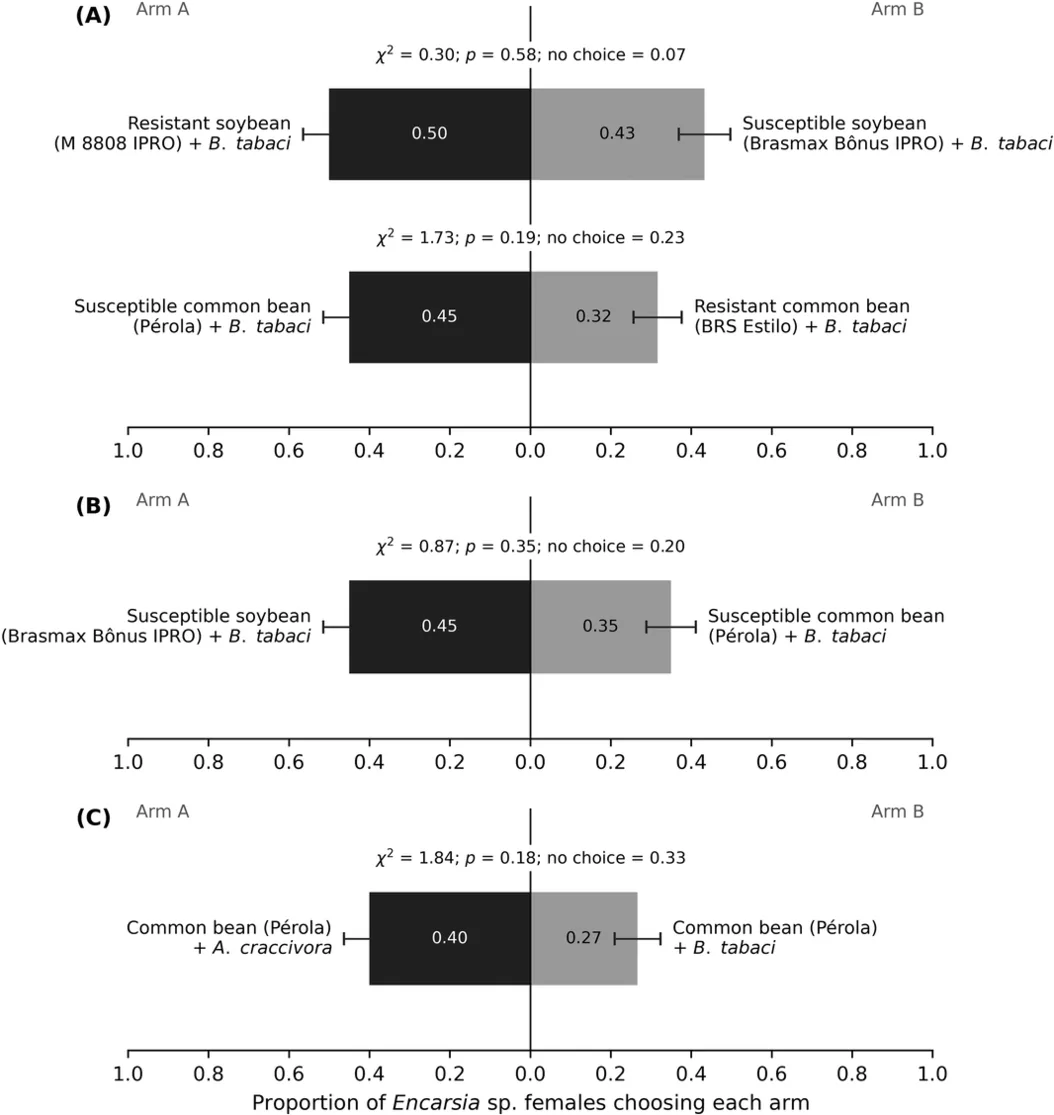
Choice responses (proportion of females choosing each arm ± SE) of *Encarsia* sp. (MATOPIBA lineage) in a Y‐tube olfactometer. (A) Resistant versus susceptible soybean (T1) and susceptible versus resistant common bean (T2), all infested with *Bemisia tabaci* MEAM1; (B) susceptible soybean versus susceptible common bean infested with *B. tabaci* (T3); (C) common bean infested with *Aphis craccivora* versus common bean infested with *B. tabaci* (T4). Arm A is shown on the left and arm B on the right, following Table 2. *n* = 60 females per contrast; the proportion of non‐responding individuals (no choice) is indicated above each pair of bars and was excluded from the chi‐square tests.

When resistant and susceptible soybean cultivars infested with *Bemisia tabaci* were compared (T1), parasitoid choice did not deviate from a 1:1 distribution (*χ* ^2^ = 0.30, *p* = 0.58; *n* = 56 responders out of 60 females). Similarly, no significant preference was observed between resistant and susceptible common bean cultivars infested with *B. tabaci* (T2; *χ* ^2^ = 1.73, *p* = 0.19; *n* = 46 responders) (Figure 2A).

In the comparison between susceptible soybean and susceptible common bean plants infested with *B. tabaci* (T3), parasitoids also showed no significant preference (*χ* ^2^ = 0.87, *p* = 0.35; *n* = 48 responders) (Figure 2B).

Likewise, when common bean plants infested with *B. tabaci* were compared to plants infested with *Aphis craccivora* (T4), no significant deviation from random choice was detected (*χ* ^2^ = 1.84, *p* = 0.18; *n* = 40 responders) (Figure 2C).

Across all contrasts, Cramér's V values indicated small effect sizes, consistent with weak or negligible treatment effects.

The proportion of individuals classified as “no choice” differed significantly among contrasts (*χ* ^2^ = 13.24, df = 3, *p* = 0.004), being lowest in the soybean cultivar contrast (T1: 4 of 60 females, 7%) and highest in the aphid versus whitefly contrast (T4: 20 of 60, 33%), with intermediate values in T2 (14 of 60, 23%) and T3 (12 of 60, 20%); the three latter contrasts did not differ from each other (*χ* ^2^ = 3.04, df = 2, *p* = 0.22). Because no‐choice individuals were excluded from the preference analyses, this heterogeneity does not affect the choice results, but it indicates that overall responsiveness varied with the odour context. In all contrasts, the proportion of responding females (67%–93%) was considerably higher than in the blank test (36.7%).

### 3.2 Residence Time

Residence time did not differ significantly among treatment combinations in any of the olfactometer zones (Figure 3).

**Figure 3:**
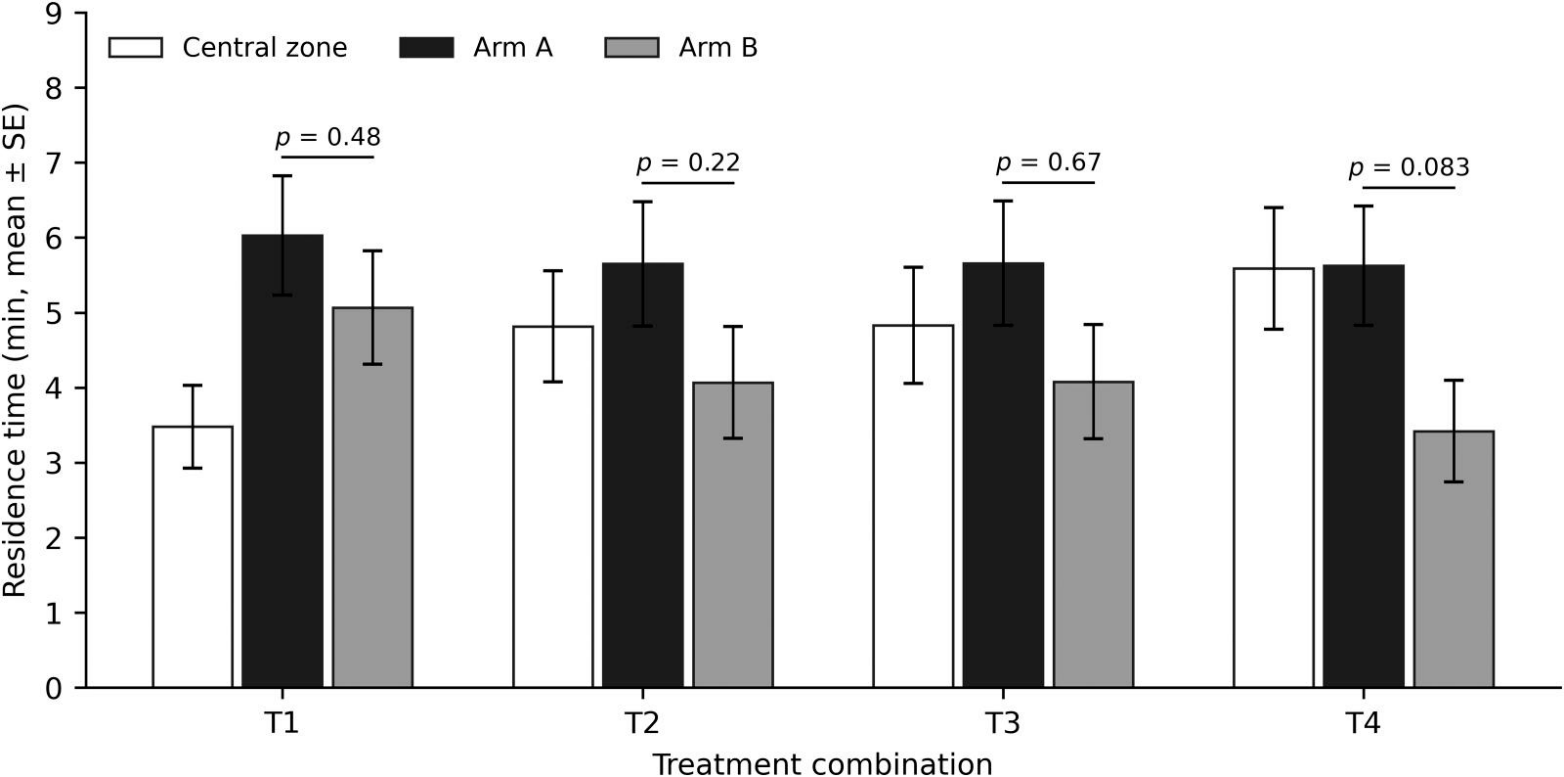
Residence time (min, mean ± SE) of *Encarsia* sp. (MATOPIBA lineage) females in the central zone, arm A and arm B of the Y‐tube olfactometer for each treatment combination (*n* = 60 females per combination). T1: resistant soybean M 8808 IPRO + *Bemisia tabaci* (arm A) versus susceptible soybean Brasmax Bônus IPRO + *B. tabaci* (arm B); T2: susceptible common bean Pérola + *B. tabaci* (arm A) versus resistant common bean BRS Estilo + *B. tabaci* (arm B); T3: susceptible soybean Brasmax Bônus IPRO + *B. tabaci* (arm A) versus susceptible common bean Pérola + *B. tabaci* (arm B); T4: common bean Pérola + *Aphis craccivora* (arm A) versus common bean Pérola + *B. tabaci* (arm B). *p*‐values above the bars refer to Wilcoxon signed‐rank tests comparing arm A and arm B within each combination. Kruskal–Wallis tests among combinations: central zone, H = 0.774, *p* = 0.856; arm A, H = 1.020, *p* = 0.796; arm B, H = 3.633, *p* = 0.304.

In the central zone, no significant differences were detected among treatments (Kruskal–Wallis: *H* = 0.774, *p* = 0.8557). Mean residence time ranged from 3.47 to 5.59 min (Figure 3).

Similarly, no differences were observed for time spent in arm A (*H* = 1.020, *p* = 0.7964), where values ranged from 5.62 to 6.03 min, or in arm B (*H* = 3.633, *p* = 0.3039), with residence times varying from 3.42 to 5.06 min (Figure 3).

Within each treatment combination, the time spent in arm A did not differ significantly from the time spent in arm B (Wilcoxon signed‐rank test; T1: *W* = 764.0, *p* = 0.48, *r* = 0.09; T2: W = 511.5, *p* = 0.22, *r* = 0.16; T3: W = 523.5, *p* = 0.67, *r* = 0.06) (Figure 3). In T4, females tended to spend more time in the arm carrying volatiles from *A. craccivora*‐infested plants (mean ± SE: 5.63 ± 0.79 min) than in the arm with *B. tabaci*‐infested plants (3.42 ± 0.68 min), but this difference did not reach statistical significance (*W* = 346.5, *p* = 0.083, *r* = 0.22).

Effect size estimates (*η* ^2^) for residence time analyses were low across all contrasts, indicating that treatment factors explained only a minimal proportion of behavioural variance.

## 4 Discussion

The absence of significant directional responses across all contrasts indicates that the MATOPIBA *Encarsia* lineage did not exhibit detectable long‐range olfactory discrimination between resistant and susceptible cultivars, between host plant species, or between different herbivore infestations under the conditions tested. These findings suggest that variation in plant resistance status and herbivore identity did not generate sufficiently contrasting volatile cues to elicit consistent directional orientation in the Y‐tube system.

An important methodological caveat must be acknowledged when interpreting these results. Because the objective of the study was to compare the relative attractiveness of paired plant–herbivore combinations, all contrasts were run as plant‐versus‐plant comparisons and no positive control (e.g., whitefly‐infested plant vs. clean air) was included. Consequently, the absence of discrimination cannot be unequivocally separated from a low overall responsiveness of this lineage to plant volatiles in the Y‐tube system, even though blank (air vs. air) trials confirmed the absence of positional bias, and the proportion of females that actively entered one of the arms was markedly higher in all plant contrasts (67%–93%) than in the blank test (37%), indicating that plant volatiles stimulated upwind movement even though they did not elicit directional preference. Responsiveness also varied among contrasts, being highest when the two soybean cultivars were compared and lowest when aphid‐ and whitefly‐infested plants of the same cultivar were compared, which suggests that the overall stimulatory effect of the odour pairs differed even in the absence of discrimination. In addition, in a previous study with the same *Encarsia* lineage, the same air‐delivery system and odour‐source chambers and the same soybean cultivars, infested plants were tested against an empty chamber (Silva et al. 2025): females showed a detectable, although modest, response to *B. tabaci*‐infested Bônus plants (longer residence in the arm carrying plant volatiles and a markedly greater distance travelled), but no attraction to infested M 8808 plants. Those results indicate that the system is capable of revealing responses to plant volatiles in this lineage, while also showing that its long‐range responses are inherently weak, a pattern fully consistent with the present findings. Moreover, when two plant odour sources are presented simultaneously, background volatiles from one plant may interfere with orientation towards the other, potentially masking weak preferences (Turlings and Erb 2018). Testing each plant–herbivore combination against a clean‐air control, or using four‐arm olfactometers or wind‐tunnel assays, would allow the intrinsic attractiveness of each odour source to be quantified and constitutes a priority for future work.

Importantly, lack of behavioural discrimination does not imply absence of volatile induction. Herbivore‐induced plant volatiles (HIPVs) are complex, dynamic blends whose ecological function depends not only on their presence but also on their concentration, relative ratios, temporal stability, and reliability as host‐location cues (Clavijo McCormick et al. 2012; Turlings and Erb 2018; Ali et al. 2023). Parasitoid attraction is often sensitive to subtle quantitative and qualitative differences in volatile composition, and even chemically distinct blends may be perceived as functionally similar if they fall within the sensory resolution threshold of the insect (Aartsma et al. 2017). Thus, the lack of preference observed here may reflect quantitative subtlety or qualitative overlap among treatments rather than absence of plant‐mediated signalling.

The absence of discrimination between soybean and common bean plants, as well as between plants infested by *B. tabaci* MEAM1 and the non‐host *A. craccivora*, further supports the possibility of volatile overlap or insufficient signal divergence. Previous studies have demonstrated that parasitoid host‐finding efficiency declines when volatile blends are ambiguous or when induction by non‐host herbivores masks host‐associated cues (de Rijk et al. 2013; de Rijk et al. 2016; Aartsma et al. 2017). In multitrophic systems, background odours and chemically heterogeneous landscapes can reduce signal contrast, limiting the effectiveness of long‐range olfactory orientation (Turlings and Erb 2018). In this context, the behavioural pattern observed here may reflect sensory constraints rather than absence of tritrophic communication.

In addition, this absence of olfactory preference of *Encarsia* sp. MATOPIBA lineage may also be associated with the previous experience of these individuals, since they were raised on kale before the tests. Van Wijk et al. (2008) and Gonthier et al. (2023) suggest that natural enemies may adjust their olfactory responses based on learning, becoming more sensitive to odours previously associated with their host. Although rearing on a plant species different from those tested avoided pre‐exposure to the test‐plant volatiles, it also means that females lacked any associative experience with soybean or common bean odours, which may reduce responsiveness to these cues relative to females reared on the same crops (Ayelo et al. 2021). Nevertheless, kale‐reared females of this lineage readily recognised and attacked *B. tabaci* nymphs on soybean leaf discs in a previous study (Silva et al. 2025), indicating that the lack of experience with the test plants does not prevent host recognition at short range. Thus, it is possible that previous rearing history influenced the olfactory responsiveness of *Encarsia* sp. under the experimental conditions evaluated. Rearing the parasitoids on the crop species of interest, or providing an oviposition experience on infested test plants before the assays, should therefore be considered in future studies.

Residence time analyses reinforce this interpretation, the similar time allocation across olfactometer arms and central zones suggests exploratory behaviour without consistent directional commitment. Within‐treatment comparisons confirmed that, once in the arms, females did not allocate significantly more time to either odour source, although a non‐significant trend towards longer residence in the arm carrying *A. craccivora*‐infested plants was observed in T4. This trend parallels the slightly higher (but also non‐significant) initial choice for this odour source and may deserve further investigation. Such patterns are frequently associated with low signal contrast or generalist search strategies in parasitoids operating under chemically heterogeneous conditions. Parasitoid host location typically involves sequential decision‐making and integration of multiple sensory modalities, including olfactory, visual, and contact cues (Ayelo et al. 2022). When long‐range volatile signals are weak or insufficiently discriminatory, short‐range stimuli such as honeydew, contact kairomones, or host‐derived tactile cues may assume greater importance (Ayelo et al. 2022; Silva et al. 2025).

An additional consideration relates to herbivore developmental stage. In this study, plants were primarily infested with *B. tabaci* nymphs and eggs (adults having been removed before the assays), which may differ from adults in their capacity to induce volatile emissions. Notably, Chen et al. (2020) showed that *E. formosa* is attracted to tomato plants infested by *B. tabaci* adults but not to plants infested only by nymphs, and the same lineage studied here showed weak responses to nymph‐infested soybean plants in a previous study (Silva et al. 2025). Variation in herbivore feeding stage and density can significantly alter the quantity and composition of HIPVs, influencing parasitoid responsiveness (Turlings and Erb 2018; Chen et al. 2020). Therefore, the limited behavioural differentiation observed here may partially reflect differences in induction intensity rather than absence of plant response.

The findings are consistent with a flexible foraging strategy in which long‐range orientation may not rely strongly on cultivar‐specific volatile differences. Instead, parasitoids may exhibit context‐dependent responsiveness, adjusting their reliance on olfactory cues according to signal reliability and environmental heterogeneity. Such plasticity has been widely documented in parasitoid sensory ecology and may represent an adaptive strategy to cope with spatial and temporal variability in host‐associated signals (Clavijo McCormick et al. 2012; Aartsma et al. 2017).

Lineage‐specific behavioural variation should also be considered. The *Encarsia* population studied here represents an undescribed MATOPIBA lineage, and behavioural divergence among closely related species is well documented. Differences in olfactory sensitivity, host range breadth, and ecological specialization may shape how distinct lineages respond to volatile cues (Li et al. 2014; Chen et al. 2020; Saranya and Kennedy 2022). Therefore, the absence of discrimination observed here should not be generalized across the genus.

From an applied perspective, the lack of differential responses between resistant and susceptible cultivars suggests that host‐plant resistance traits do not interfere with parasitoid orientation at the long‐range scale evaluated. This may be advantageous in integrated pest management systems, as varietal deployment is unlikely to reduce parasitoid attraction under comparable ecological conditions. However, the context‐dependent limits of volatile‐mediated tritrophic signalling observed here highlight the importance of integrating behavioural assays with chemical characterization of volatile blends and electrophysiological analyses to better understand signal detectability and functional thresholds (Turlings and Erb 2018).

Preliminary headspace GC–MS analyses carried out by our group indicated that the volatile profiles of *B. tabaci*‐infested Bônus and M 8808 plants differ, with the resistant cultivar forming a distinct cluster in multivariate analyses, whereas differences between intact and infested Bônus plants were subtle (Silva 2025). These preliminary data suggest that the absence of discrimination in T1 did not result from identical blends, but rather from differences that were not behaviourally resolved at long range, and that induction by nymph‐stage feeding may be weak. A full characterisation, combining headspace collection with GC–MS and GC–EAD analyses of all the cultivars and herbivore combinations tested here, would reveal whether resistance status and herbivore identity actually alter emission intensity and blend composition, which is a prerequisite for a mechanistic interpretation of the behavioural results. Direct comparison of the MATOPIBA lineage with a well‐characterized species such as *E. formosa*, which responds to volatiles from whitefly‐infested plants (Birkett et al. 2003; Chen et al. 2020), under identical assay conditions would further clarify whether the limited discrimination observed here reflects lineage‐specific sensory traits or properties of the plant–herbivore systems evaluated. Integrating such chemical and comparative approaches with electrophysiological analyses will be essential to determine whether the absence of behavioural discrimination reflects signal overlap, low emission intensity or sensory limitations of this lineage.

In summary, under controlled olfactometer conditions, long‐range orientation of the MATOPIBA *Encarsia* lineage was not detectably shaped by plant resistance status, host plant species, or herbivore identity. Rather than indicating absence of plant‐mediated signalling, these findings suggest that volatile contrasts among the evaluated crop systems were insufficient to elicit consistent directional responses at the spatial scale tested. By identifying ecological contexts in which HIPVs fail to generate discriminatory parasitoid responses, this study advances understanding of sensory constraints, signal reliability, and lineage‐specific behavioural adaptation in tritrophic interactions.

## Author Contributions

**Daniel Marques Pacheco:** investigation, writing – original draft, methodology, visualization, formal analysis. **Maria Carolina Farias e Silva:** writing – review and editing, validation, formal analysis. **Adriele Castro Ferreira:** writing – original draft, methodology, validation, visualization, software, formal analysis. **Matheus Monteiro de Santana:** validation, visualization, formal analysis. **Rodolfo Molinário de Souza:** writing – review and editing, software, formal analysis. **Jenilton Gomes da Cunha:** investigation, software, formal analysis. **Alexandre Igor de Azevedo Pereira:** writing – review and editing, formal analysis, software. **Bruno Ettore Pavan:** writing – review and editing, software, formal analysis, supervision. **José Bruno Malaquias:** software, formal analysis, writing – review and editing, supervision. **Ana Paula Gonçalves da Silva Wengrat:** formal analysis, software, writing – review and editing, visualization, validation. **Luciana Barboza Silva:** conceptualization, investigation, funding acquisition, writing – original draft, supervision, resources.

## Conflicts of Interest

The authors declare that they have no competing interests or financial conflicts of interest regarding this work.

## Data Availability

Data sharing not applicable to this article as no datasets were generated or analysed during the current study.
